# Synergistic Effect of 300 μm Needle-Depth Fractional Microneedling Radiofrequency on the Treatment of Senescence-Induced Aging Hyperpigmentation of the Skin

**DOI:** 10.3390/ijms22147480

**Published:** 2021-07-13

**Authors:** Young In Lee, Eunbin Kim, Dong Won Lee, Jemin Kim, Jihee Kim, Won Jai Lee, Ju Hee Lee

**Affiliations:** 1 Department of Dermatology, Severance Hospital, Cutaneous Biology Research Institute, Yonsei University College of Medicine, Seoul 03722, Korea; ylee1124@yuhs.ac (Y.I.L.); DGHKL17@yuhs.ac (E.K.); JEMIN89ZZ@yuhs.ac (J.K.); mygirljihee@yuhs.ac (J.K.); 2 Scar Laser and Plastic Surgery Center, Yonsei Cancer Hospital, Yonsei University College of Medicine, Seoul 03722, Korea; 3 Department of Plastic and Reconstructive Surgery, Severance Hospital, Yonsei University College of Medicine, Seoul 03722, Korea; XYPHOSS@yuhs.ac (D.W.L.); PSWJLEE@yuhs.ac (W.J.L.)

**Keywords:** lentigo, melasma, fractional microneedling radiofrequency, Nd:YAG laser, ultraviolet B, senescence

## Abstract

Aging-associated dermatological pigmentary diseases are associated with accumulation of senescence cells and the disruption of basement membrane due to chronic ultraviolet radiation (UVR) exposure. Our study is on the synergistic effect of the novel 300 μm needle-depth fractional microneedling radiofrequency (FMR) treatment and conventional Q-switched ND:YAG laser on aging-associated hyperpigmentation of the skin. The prospective controlled clinical trial of 25 Asian women revealed significantly higher improvements not only on wrinkles, but also on hyperpigmentation. Additional ex vivo study revealed significant reduction of pro-melanogenic markers as well as senescent keratinocytes, while increased expression of collagen type IV on the epidermal basement membrane, after additional FMR treatment on UV-irradiated human tissues. These results demonstrate that 300 μm needle-depth FMR might effectively remove senescent keratinocytes that secrete pro-melanogenic markers, and repair disrupted basement membrane, therefore preventing constant hyperpigmentation of the aged skin.

## 1. Introduction

Human skin, unlike other organs, undergoes photoaging processes in addition to natural aging, which ultimately leads to aging-associated pigmentary changes. The acquired benign hyperpigmentation disorders associated with aging include a wide range of diseases such as lentigo, melasma, and post-inflammatory hyperpigmentation (PIH) [1]. Among them, senile lentigo is caused by a chronic exposure to ultraviolet radiation (UVR), resulting in a characteristic hyperpigmented basal epidermal layer [2]. Hyperpigmentation in melasma, which is also affected by UVR, is frequently described as a manifestation of skin cellular senescence and is attributed to a consequent decrease in the physiological regulation of melanogenic biosynthetic pathways [3]. One of the earliest signs of melasma in the Asian population presents with mixed epidermal and dermal hyperpigmentation, showing resistance to treatment with conventional lasers, such as Q-switched (QS) or long-pulsed nanosecond neodymium-doped yttrium aluminum garnet (Nd:YAG) laser [3,4].

A recent meta-analysis on laser and laser compound therapy for melasma reported that the two most commonly used treatment modalities for aging-associated facial pigmentation until 2018 had been 1064 nm Nd:YAG and 1550 nm non-ablative fractional lasers [5]. The QS lasers targeting the destruction of melanocytes, including 532 nm QS Nd:YAG (QSNY), ruby, and alexandrite lasers, have been popular in treating not only melasma, but also senile lentigo; however, frequent occurrences of PIH, appearing in 10–25% of the cases, were reported post-treatment [6,7,8]. Meanwhile, Iranmanesh et al. [8] (2021) recently published a review article on the efficacy of energy-based devices combination therapy for melasma and stated that fractional microneedling radiofrequency (FMR) was a novel adjuvant treatment for melasma treated with low-fluence QSNY laser.

Abnormalities in the extracellular matrix, basement membrane disruption, and an increase in the vascularity and number of mast cells, in addition to epidermal pigmentation, are frequently observed on histopathological analysis of melasma [9,10,11,12]. Kwon et al. [13] (2019) demonstrated in their retrospective review that a combination therapy with FMR showed a better efficacy in treating melasma than the conventional QSNY monotherapy. The study emphasized that while black chromophore-targeting laser treatments could produce considerable degrees of unwanted side effects, such as mottled hypopigmentation and rebound hyperpigmentation, combination therapy with FMR reduced the incidence of these side effects by stabilizing the activity of melanocytes and the function of basement membrane [13]. The aim of this study was to investigate the efficacy of 300 μm needle-depth FMR on aging-associated hyperpigmentation of the face via affecting epidermal cellular senescence. We performed a prospective clinical trial on 25 Asian women to observe the synergistic effect of 300 μm needle-depth FMR and conventional laser toning on aging-associated hyperpigmentation. An additional human ex vivo study was conducted to explore the pathophysiologic mechanism by which FMR treatment resulted in skin lightening.

## 2. Results

### 2.1. Patients

Twenty-five Asian women with facial pigmentation due to photoaging were enrolled in the present study. The mean age of the subjects was 51.4 ± 7.56 years (range, 39–63 years). Among the patients, 14 had Fitzpatrick skin type III and 11 had Fitzpatrick skin type IV. In addition, 11 patients had a single type of pigmentation, melasma, while 14 presented with a mixed type of age-related hyperpigmentation, including melasma and senile lentigo.

### 2.2. Clinical Efficacy of Adjuvant FMR Treatment after QSNY for Aging-Associated Hyperpigmentation

**Figure 1:**
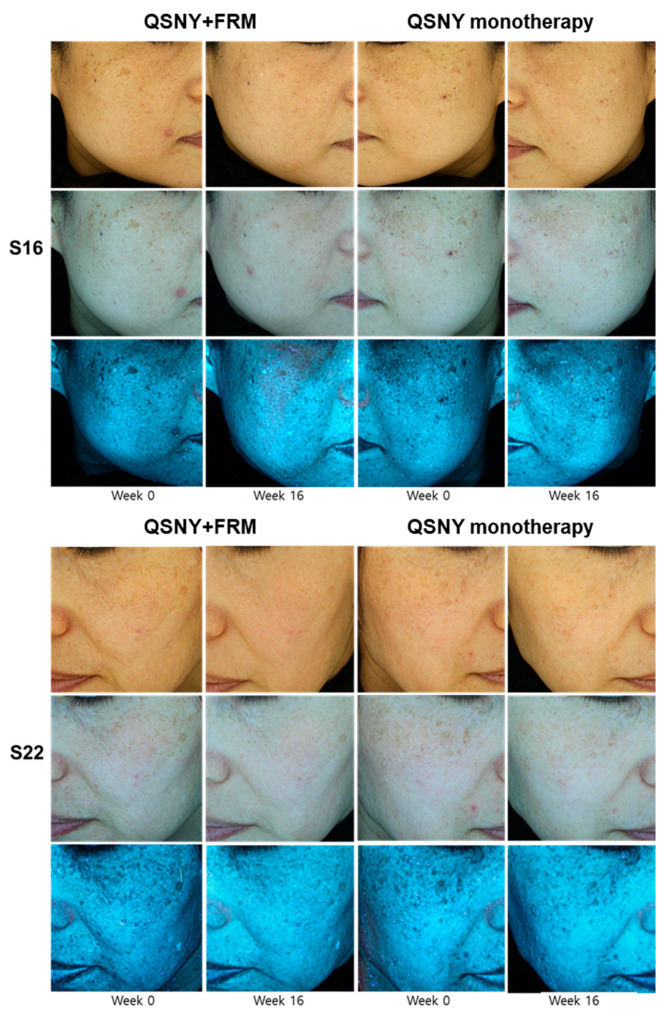
The comparison of the improvements of aging-associated facial pigmentation between the QSNY + FMR group and QSNY group at baseline and week 16.

**Figure 2:**
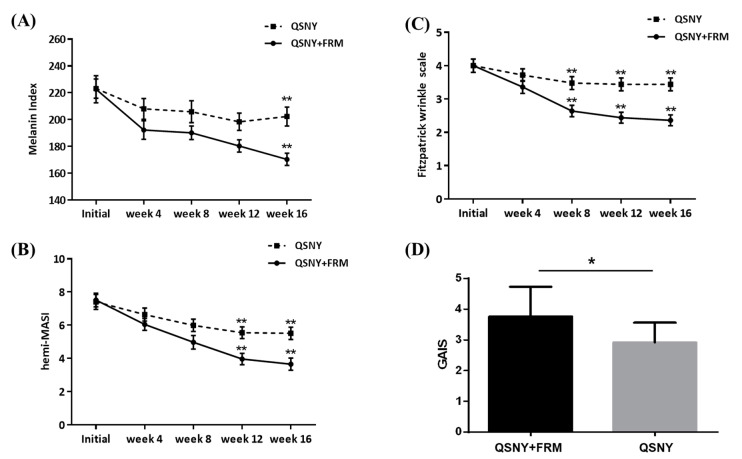
Clinical efficacy of adjuvant FMR treatment after QSNY for aging-associated hyperpigmentation. Improvements on pigmentation were assessed both (**A**) quantitatively by the melanin index and (**B**) qualitatively by hemi-MASI. (**C**) Improvements on wrinkles were evaluated via the Fitzpatrick wrinkle and elastosis scale. (**D**) GAIS was assessed by the investigators on week 12. Post hoc analyses for multiple comparison after RM-ANOVA on melanin index and hemi-MASI were performed via Student’s *t*-test after assuming normality (Kolmogorov–Smirnov test, *p* > 0.05). Statistical analysis for multiple comparison on Fitzpatrick wrinkle scale and GAIS were performed via Mann–Whitney *U* test. * *p* < 0.05, ** *p* < 0.01, respectively.

The additive effect of FMR with QSNY on age-related hyperpigmentation was evaluated both quantitatively and qualitatively, by measuring the melanin index, Hemi Melasma Area and Severity index (hemi-MASI), Fitzpatrick wrinkle and elastosis scale, and Global Aesthetic Improvement Scale (GAIS). The QSNY + FMR group showed a significantly higher reduction in the melanin index during the study period than the QSNY group (*p* = 0.039, Figure 1 and Figure 2A). Multiple comparison post hoc analyses revealed that the QSNY + FMR group showed a significantly lower melanin index at week 16 than the QSNY group (*p* < 0.01). Irrespective of the treatment methods, a significant reduction in the hemi-MASI was observed in both groups (*p* < 0.05). Nonetheless, the QSNY + FMR group showed a significantly higher reduction in the hemi-MASI than the QSNY group at weeks 12 and 16 (*p* < 0.01, Figure 2B). In addition, the improvement in wrinkles was assessed, and the QSNY + FMR group showed a significantly higher reduction in the Fitzpatrick Wrinkle and Elastosis Scale than the QSNY group (*p* < 0.001, Figure 2C). Multiple comparison post hoc analyses revealed that the QSNY + FMR group showed a significantly lower Fitzpatrick Wrinkle and Elastosis Scale at weeks 8, 12, and 16 than the QSNY group (*p* < 0.01). Finally, the global improvements evaluated by the blinded, independent investigators revealed a significantly higher GAIS in the QSNY + FMR group than in the QSNY group (*p* < 0.05, Figure 2D).

### 2.3. Safety Profile

Pain, post-treatment erythema, and crusting were well tolerated by all the patients who had undergone repeated sessions. Serious adverse effects, including scarring, textural changes, bleeding, and secondary infections, were not observed during any treatment session or the follow-up period. The patients reported much less pain and erythema after QSNY + FMR (300 μm needle-depth) treatment compared with the degree of pain and erythema after conventional FMR alone.

### 2.4. Effect of FMR Irradiation after UVB Exposure on Skin Pigmentation

**Figure 3:**
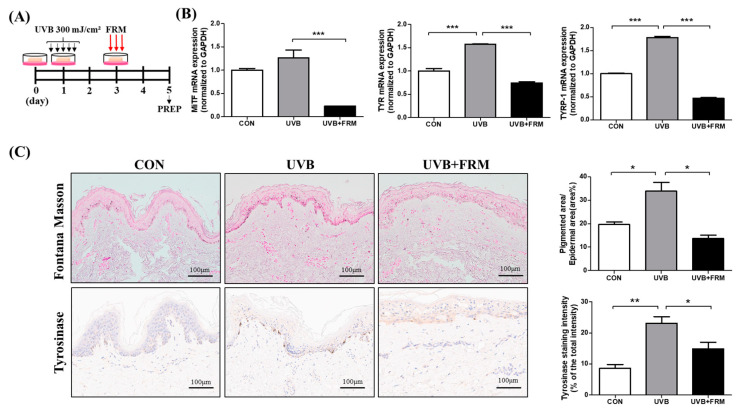
Effect of FMR irradiation after UVB exposure on skin pigmentation. (**A**) Ex vivo human skin study schedule. (**B**) qRT-PCR results show decreased pro-melanogenesis markers, MITF, TYR, and TYRP-1, in UVB-irradiated skin after FMR. (**C**) Fontana–Masson staining and tyrosinase IHC staining show significant reduction of pigmented area% and the tyrosinase activity of UVB-irradiated skin tissue after FMR. Statistical analyses were performed via Mann–Whitney *U* test. * *p* < 0.05, ** *p* < 0.01, *** *p* < 0.005, respectively.

To further assess the effect of FMR on aging-associated hyperpigmentation, we designed a human ex vivo study and irradiated the skin samples with UVB (Figure 3A). Fontana–Masson staining revealed a significant increase in basal epidermal pigmentation after UVR, while subsequent FMR treatments significantly decreased the degree of epidermal pigmentation (*p* < 0.05, Figure 3B,C). Moreover, mRNA expression levels of markers of melanogenesis, such as microphthalmia-associated transcription factor (MITF), tyrosinase (TYR), and tyrosinase-related protein 1 (TRP-1), were significantly reduced after UVB + FMR treatment compared with those after UVB treatment alone, indicating a possible skin-lightening role of FMR on photoaged, pigmented skin (*p* < 0.005, Figure 3C).

### 2.5. UVB-Induced Premature Senescence of Epidermal Keratinocytes Rescued by FMR Irradiation

**Figure 4:**
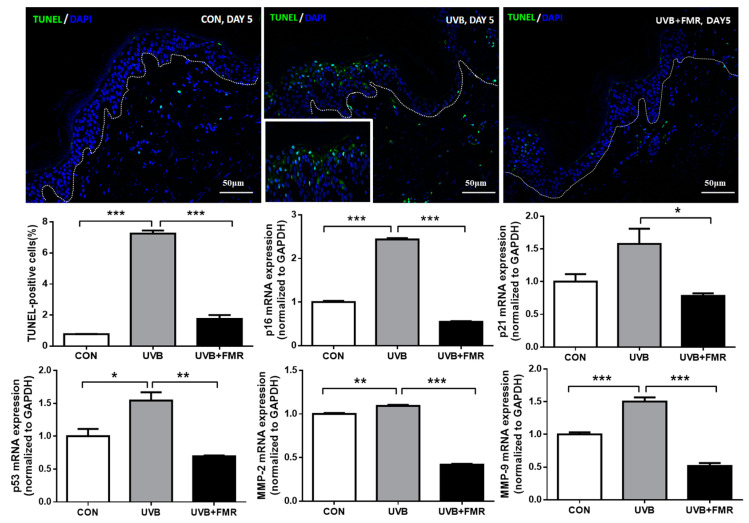
Effect of FMR irradiation after UVB exposure on epidermal cellular senescence. The TUNEL assay shows significantly decreased TUNEL-positive cells after FMR on UVB–irradiated skin. qRT-PCR analyses of premature senescence markers, including p16, p21, p53, MMP-2, and MMP-9 show significant reductions after FMR on UVB-irradiated skin. Statistical analyses were performed via Mann–Whitney *U* test. * *p* < 0.05, ** *p* < 0.01, *** *p* < 0.005, respectively.

To investigate the molecular mechanism by which 300 μm needle-depth FMR resulted in skin lightening, we performed terminal deoxynucleotidyl transferase-mediated dUTP nick end-labeling (TUNEL) assay to analyze genomic instability and premature senescence induced by UVR. Our ex vivo study demonstrated that the number of TUNEL-positive epidermal cells increased after UVB irradiation, whereas subsequent FMR treatment decreased their numbers (Figure 4, *p* < 0.005). Stress-induced premature senescence is a genetically controlled process, mediated by p16 and p21, depending on the cellular p53 status [14]. Additional quantitative reverse transcription–polymerase chain reaction (qRT–PCR) revealed that the mRNA expression levels of not only p16, p21, and p53, but also of matrix metalloproteinase (MMP)-2 and MMP-9 significantly increased after UVB exposure. These markers of cellular senescence concordantly decreased significantly after FMR irradiation (Figure 4, *p* < 0.05).

**Figure 5:**
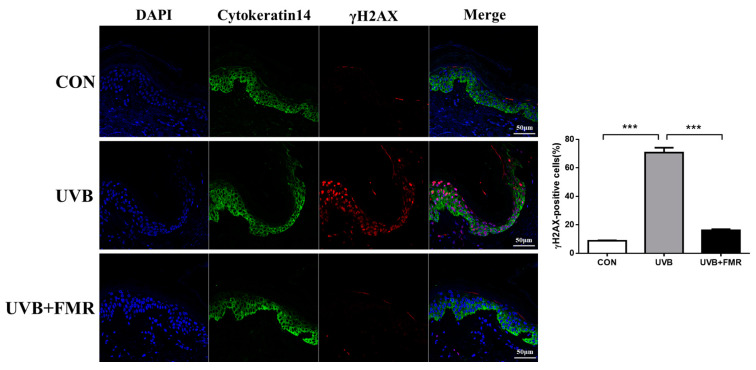
Effect of FMR irradiation after UVB exposure on DNA damage response (DDR) and keratinocyte senescence. Double immunofluorescence staining of cytokeratin 14 and γ-H2AX shows a significant reduction of DDR on UVB-irradiated epidermal keratinocytes after FMR. Statistical analyses were performed via Mann–Whitney *U* test. *** *p* < 0.005.

Previous research has shown causal connection between DNA damage response (DDR) and stress-induced cellular senescence [15]. We further analyzed the levels of proteins involved in DDR in UVB-irradiated human skin specimens, before and after the additional treatment with FMR, by staining tissues with γ-H2AX (part of DNA repair foci). The UVB-irradiated skin specimen showed significantly increased γ-H2AX positivity in cytokeratin 14-positive epidermal keratinocytes compared with the control group, while the subsequent FMR irradiation reversed this process (Figure 5, *p* < 0.005). Hence, we hypothesized that the decreased senescence signaling in the epidermis after FMR might have blocked melanogenesis in the basal melanocytes, thus augmenting skin lightening.

### 2.6. Restored Expression of Collagen Type IV in the UV-Irradiated Basal Membrane after FMR

**Figure 6:**
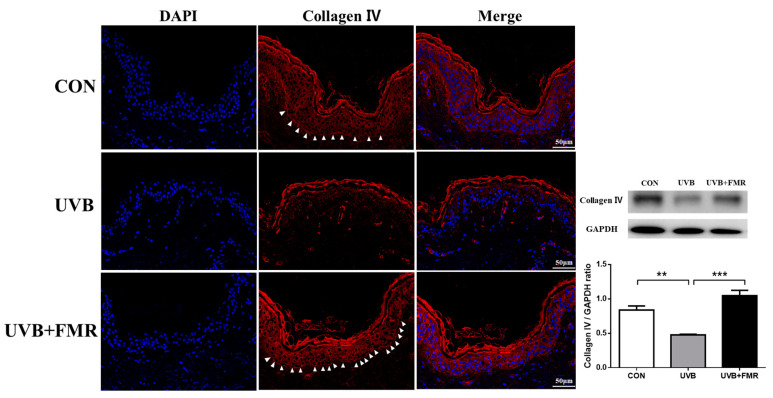
Effect of FMR irradiation after UVB exposure on collagen type IV expressed on the basement membrane. The immunofluorescence staining and the Western blot analysis of collagen type IV on ex vivo human skin reveal a significant increase in collagen type IV expression after FMR, followed by UVB irradiation (arrowheads indicates collagen type IV expression of the epidermal basement membrane). Statistical analyses were formed via Mann–Whitney *U* test. ** *p* < 0.01, *** *p* < 0.005, respectively.

Previous research has shown that the melasma tissue specimens have a disrupted basement membrane and reduced expression of type IV collagen [9]. Our ex vivo study revealed decreased expression of collagen type IV after UVB irradiation; nonetheless, FMR irradiation significantly restored the expression of collagen type IV in the basement membrane (Figure 6, *p* < 0.005). These results suggest that the increased number of senescent keratinocytes in the lower epidermis due to UVR leads to the disruption of basement membrane due to the secretion of proteases such as MMP-2 and MMP-9. This is reversed by FMR irradiation that reduces the number of senescent cells.

## 3. Discussion

Several dermatological pigmentary diseases are associated with the accumulation of senescent cells in the skin. Recent studies on aging-associated hyperpigmentation suggest that senescent fibroblasts influence epidermal melanocytes via cross-talk that occurs through the damaged basement membrane [16,17]. The disruption of the basement membrane can be partially due to the significantly upregulated expressions of MMP-2 and MMP-9 when the epidermal cells undergo cellular senescence due to chronic UVR exposure [18]. Studies on the pathogenesis of melasma suggested that the increase in MMP expression upon chronic sun exposure increased the permeability of the basement membrane [12,19]. Interestingly, the rise in MMP-2 and reduction in collagen type IV, which is the major component of the epidermal basement membrane, correlated with the increase in the number of melanocytes that protruded into the dermis in melasma [19].

A study by Yoon et al. [20] (2018) on senile lentigo demonstrated that the senescent fibroblasts upregulate MITF and tyrosinase in melanocytes via the dysregulation of stromal cell-derived factor-1 (SDF-1) signaling. They also revealed that the restoration of SDF-1 signaling after the conventional needle-depth FMR treatment was associated with skin lightening [20]. It is known that multiple types of cells of the skin, including dermal fibroblasts, epidermal keratinocytes, and melanocytes, drive skin pigmentation during the process of cellular senescence. Among them, keratinocytes are the main type of cells that are involved in the initiation of melanogenesis in the skin. Due to chronic UVR exposure, senescent keratinocytes secrete α-melanocyte stimulating hormone, endothelin (ET)-1, and stem cell factor in a p53-proopiomelanocortin (POMC)-dependent manner; the subsequent activation of MITF then plays a critical role in the cascade involved in the induction of melanogenesis [3,21,22,23].

Our prospective, randomized split-face clinical study of 25 women with aging-associated facial hyperpigmentation revealed that 300 μm needle-depth FMR treatment after conventional QSNY laser resulted in a synergistic effect on skin lightening. Compared with the QSNY monotherapy, additional treatment with 300 μm FMR after QSNY laser toning resulted in significantly higher improvement in not only the wrinkle assessment (Fitzpatrick wrinkle and elastosis scale), but also in quantitative (melanin index) and qualitative (hemi-MASI) pigmentation assessments. The overall GAIS score was also higher in the QSNY + FMR group. FMR is currently being used for multiple indications, such as wrinkles and skin tightening, acne scars, stretch marks, hair thinning, and rosacea/post-inflammatory erythema [24]. Our study shows that non-insulated FMR with the novel 300 μm needle-depth approach can affectively target the epidermal basement membrane with minimal pain, which can lead to skin lightening. Our ex vivo study results correlated with the clinical findings, showing reduced basal pigmentation on Fontana–Masson staining as well as decreased mRNA expressions of pro-melanogenic markers (MITF, TYR, and TRP-1) after UVB + FMR exposure compared with UVB exposure alone.

The mechanism of clinically significant synergistic effect on the reduction of aging-associated pigmentation with 300 μm needle-depth FMR needs further investigation. Cui et al. (2007) had shown that UV induction of POMC/MSH in skin is directly controlled by p53 [25]. Another study showed that the administration of p53 inhibitor suppressed the expressions of paracrine cytokines, SCF and ET-1, as well as melanogenic factors such as KIT, ETR, melanocortin 1 receptor (MC1R), MITF, and tyrosinase, resulting in inhibition of hyperpigmentation [26]. Our ex vivo study results suggested that the FMR treatment of human skin tissue effectively reduced the numbers of TUNEL- and γ-H2AX-positive keratinocytes in the epidermis. The mRNA expression of p16, p21, and p53, which are the common markers of cellular senescence, also decreased significantly after FMR. Subsequent analysis for MMP-2 and MMP-9 also showed a decrease in their levels of mRNA expression, suggesting a possible role of 300 μm FMR in removing senescent keratinocytes from the epidermis, thus preventing the initiation of p53-POMC-MITF dependent cascade of melanogenesis.

Moreover, previous studies had shown decreased expression of collagen type IV in human melasma tissues [12]. In this study, the immunofluorescence staining before and after the FMR treatment of UVB-irradiated human skin showed significantly increased linear expression of collagen type IV in the basement membrane compared with its expression in the UVB-irradiated tissues that had not undergone FMR. This suggests that 300 μm needle-depth FMR may have a role not only in removing senescent keratinocytes that are pro-melanogenic, but also in repairing the basement membrane disruption of photoaged skin, preventing the constant hyperpigmentation due to the fall of melanin into the dermis.

The limitations of our studies include the relatively small number of the sample size and the follow-up period. Moreover, there are multiple local factors other than p53-induced melanogenesis regarding UVR-induced aging-associated hyperpigmentation. Not only melanocortin/MC1R complex, but also other important regulators of melanin pigmentation, including ETs, histamine, eicosanoids, and SCF, interact with cell surface receptors to cause melanogenesis [27]. UVB also enhance the levels of corticotropin-releasing hormone, endorphin, adrenocorticotropic hormone, sex steroids, and vitamin D, thereby modifying cutaneous pigmentation [28,29]. Lastly, the p53-POMC axis discussed in this study also requires additional confirmation, as other previous studies mention its weakness with the experimental design, in particular with the use of C57BL6 mice of which the melanin pigmentation occurs independently from POMC expression [30,31]. Further in vitro studies are needed to confirm the ex vivo and clinical findings, and to provide a complete understanding of senescence-associated hyperpigmentation of the skin.

## 4. Materials and Methods

### 4.1. Clinical Study Design and Patients

We conducted a prospective, split-face, randomized, single-blinded clinical trial on 25 Asian women with aging-associated hyperpigmentation (i.e., melasma, senile lentigo, or mixed type) to evaluate the additive efficacy of FMR in reduction of facial pigmentation when combined with conventional 1064 nm Nd:YAG laser toning (QSNY + FMR vs. QSNY alone). Adult patients aged older than 30 years with Fitzpatrick skin types III to IV who presented to Severance Hospital at Yonsei University for the treatment of facial pigmentation diseases were enrolled. Exclusion criteria included history of keloid scarring, photosensitivity, uncontrolled medical illness, pregnancy, previous laser/light-based treatments, and use of cosmetics containing growth-factor-related constituents or bleaching agents during the last 6 months. The clinical trial was approved by the Institutional Review Board of Severance Hospital, Yonsei University (IRB No. 1-2020-0023, 5 Jun 2020). The protocol was initiated following written informed consent in adherence to the principles of Declaration of Helsinki.

### 4.2. Treatment Protocol

A dual-wave mode (pulsed/continuous) FMR equipped with non-insulated microneedles arranged in a 5 × 5 array (Sylfirm X^TM^; Viol Co., Ltd., Seongnam, Korea) and a 1064 nm QSNY laser (MedLite C6^TM^; HOYA ConBio Inc., Fremont, CA, USA) were used in this study. All laser treatments were performed by a single experienced dermatologist (YIL). After gentle cleansing of the skin, a topical anesthetic (2.5% lidocaine HCl and 2.5% prilocaine cream) was applied 30 min before laser treatment. The 1064 nm QSNY was delivered with a fluence ranging from 2.5 to 3.5 J/cm^2^, spot size of 6 mm, and a repetition rate of 10 Hz for three overlapping passes. Consecutively, FMR was performed using the pulsed-wave mode (PW2), 300 μm microneedle-depth, and a power level ranging from 4 to 6 for two passes on one side of the face, chosen randomly by the investigator before the laser treatment. Two treatment sessions were performed each week for a total of five weeks for each patient (weeks 0, 2, 4, 6, and 8).

### 4.3. Efficacy Evaluation

Standard clinical photographs were taken at baseline and before each treatment with a digital camera (Canon EOS 800D, Canon Inc., Tokyo, Japan) and were evaluated using a specialized digital photography analyzer (Mark Vu^TM^, PSI Plus, Suwon-si, Gyeonggi-do, Korea). In addition, the melanin index was measured using Mexameter^®^ MX 18 (Courage + Khazaka Electronic GmbH, Köln, Germany). Improvements in pigmentation and wrinkles were evaluated using the hemi-MASI score and 9-point Fitzpatrick Wrinkle and Elastosis Scale, respectively, as previously described [22,23]. Each measurement was taken at baseline and at weeks 4, 8, 12, and 16. After the end of the treatment period (10 sessions in total), all patients were followed up for 8 weeks for the evaluation of long-term safety and efficacy of the laser treatment. Three blinded investigators (JHL, JMK, JK) independently graded the QSNY + FMR and QSNY side of each patient using the GAIS at the end of the study by comparing clinical photographs taken at baseline and week 12.

### 4.4. Safety Evaluation

On each visit to the dermatology department, we performed detailed physical examination for each patient to assess the safety of the procedures. In addition, patient reports regarding the type and severity of adverse events (e.g., hyper- or hypopigmentation, pin-point bleeding, bruising, and scarring) after laser treatments were carefully documented.

### 4.5. Ex Vivo Skin Organ Culture and UVB/FMR Irradiation Protocol

Residual normal human skin specimens that were left behind during free transverse rectus abdominis myocutaneous flap breast reconstruction surgery were obtained from the Department of Plastic Surgery, after approval from the Institutional Review Board of Severance Hospital (IRB No. 4-2021-0262). Tissue specimens were divided into three groups: control, UVB, and UVB + FMR group. Tissues that were going to be exposed to UVR were cut into 2 × 2 cm^2^ pieces and irradiated twice using UVB lamps (G8T5E, Sankyo Denki, Kanagawa, Japan) emitting 306 nm radiations at a dose of 300 mJ/cm^2^ and power of 8 W, for 2 consecutive days. For the UVB + FMR group, UVB-exposed specimens were incubated for 24 h and irradiated with Sylfirm X^TM^, at 300 μm microneedle-depth, PW2 mode, and power level 4. The specimens were then cultured for 48 h and were harvested for preparation. All tissues, including those in the control group, were cultured for a total of 5 days. All experiments were repeated 3 times, independently.

### 4.6. Immunohistochemistry and Immunofluorescence

Melanin pigments were detected with a Fontana–Masson stain kit (ab150669, Abcam, Cambridge, MA, USA). An image analysis was performed using Image J software (version 1.52a; National Institutes of Health, Bethesda, MD, USA) to quantify the percentage of pigmented area out of the total epidermal area (area %).

Immunohistochemistry was performed on formalin-fixed, paraffin-embedded skin tissue sections (5 μm thick). Sections were boiled in EnV FLEX TRS High pH (50×) (GV804-11-2, DACO, Carpinteria, CA, USA) for 30 min for antigen unmasking. Slides were incubated with 3% H_2_O_2_ (7722-84-1, Junsei Chemical Co., Ltd., Tokyo, Japan) on ice for 10 min followed by blocking in 5% bovine serum albumin (BSAS 0.1, Bovogen Biologicals, Williams, East Keilor, Australia) with blocking buffer for 1 h at room temperature. After washing with tris-buffered saline with 0.1% Tween^®^ 20 detergent (TBS-T) (BTT-9120, Tech&Innovation, Chuncheon-si, gangwon-do, Korea), the sections were incubated with primary antibodies against Tyrosinase (ab738, dilution, 1:50; Abcam) overnight at 4 °C. A DAKO peroxidase/DAB detection kit (K5007, DAKO) was used for detection. Slides were stained with Mayer’s hematoxylin (s3309, DAKO) to visualize the nuclei.

For Immunofluorescence, human skin cryosections (5 μm thick) were fixed for 30 min at RT with 4% paraformaldehyde (BPP-9004, Tech&Innovation), rinsed with TBS-T (BTT-9120, Tech&Innovation) and incubated with primary antibody: γH2AX (ab81299, dilution, 1:250; Abcam, Cambridge), cytokeratin14 (ab7800, dilution, 1:100; Abcam), and collagen IV(ab6586, dilution, 1:1000; Abcam) overnight at 4 °C. Sections were washed and incubated for 1 h at room temperature with Alexa Fluor-488-conjugated anti-mouse IgG antibody (A11008, dilution, 1:1000, Thermo Fisher Scientific, Waltham, MA, USA) and Alexa Fluor-555-conjugated anti-rabbit IgG antibody (A27039, dilution, 1:1000, Thermo Fisher Scientific). After detection, the sample was washed with TBS-T and mounted using a VECTASHIELD mounting medium containing 4′,6-diamidino-2-phenylindole (DAPI) (H-1200-10, Vector Laboratories Inc., Burlingame, CA, USA). All fluorescence images were observed with a laser-scanning microscope (LSM 700, Carl Zeiss, Jena, Germany).

### 4.7. Terminal Deoxynucleotidyl Transferase-Mediated dUTP Nick End-Labeling Assay

Apoptotic cells in the skin explant tissue were detected using the in situ Cell Death Detection Kit, Fluorescein (11684795910, Roche, Penzberg, Germany) according to the manufacturer’s protocol. TUNEL assay was performed on cryosections (5 μm thick). The coverslips were mounted with VECTASHIELD mounting medium containing DAPI. The samples were observed with a laser-scanning microscope (LSM 700, Carl Zeiss, Jena, Germany) and analyzed with LSM 5 image browser software (Carl Zeiss).

### 4.8. RNA Isolation and Quantitative Reverse Transcription–Polymerase Chain Reaction

Total RNA from the skin explants was extracted using the RNAiso Plus (9109, Takara Bio, Kusatsu, Shiga Prefecture, Japan), according to the manufacturer’s instructions. RNA was quantified using NanoDrop 2000 spectrophotometer (NanoDrop Technologies, Wilmington, DE, USA). Complementary DNA (cDNA) was synthesized using the RNA to cDNA EcoDry Premix Kit (Takara Bio). qRT–PCR was performed on the solution containing SYBR Green Master Mix (4309155, Promega Co., Madison, WI, USA), and specific primer pairs (Macrogen, Seoul, Korea) using the QuantStudio 3 Real-Time PCR System (Applied Biosystems, Thermo Fisher Scientific) in 20 μL reactions. PCR primer sequences are provided in Table 1. Cycling conditions were as follows: 95 °C for 10 min, followed by 40 cycles at 95 °C for 15 s, 60 °C for 20 s, and 72 °C for 30 s. After performing qRT-PCR, the amount of mRNA was calculated by the relative quantification method. Glyceraldehyde 3-phosphate dehydrogenase (GAPDH, Cell Signaling Technology, Danvers, MA, USA) was used as a housekeeping gene for normalization of the gene expression levels. The primer sequences are proven as listed below.

### 4.9. Western Blot Analysis

The tissue samples were washed twice with cold phosphate-buffered saline and homogenized using a mechanical TissueLyser Ⅱ (Qiagen, Hilden, Germany) with stainless steel beads at 30 Hz for 2 min. Total protein was lysed with radioimmunoprecipitation assay lysis buffer (RC2002, Biosesang, Seongnam-si, Gyeonggi-do, Korea), containing 1% protease inhibitor cocktail (PPI 1015, Quartett, Berlin, Germany), and protein concentrations were quantified using a bicinchoninic acid protein assay (B9643-1L, Sigma-Aldrich). Equal amounts (20 μg) of protein for each sample were separated by sodium dodecyl sulfate-polyacrylamide gel electrophoresis (6–12% gradient gel) and transferred to nitrocellulose membranes. Membranes were blocked with 5% skim milk (#232100, BD Biosciences, Seoul, Korea) at room temperature for 1 h and then probed overnight at 4 °C with the primary antibodies against collagen Ⅳ (ab6586, dilution, 1:1000; Abcam) and GAPDH (2118S, dilution, 1:1000; Cell Signaling Technology), followed by treatment with anti-rabbit IgG secondary antibody (7074S, dilution, 1:2000; Cell Signaling Technology) for 1 h at room temperature. The protein bands were treated with enhanced chemiluminescent detection reagent (LF-QC0101, Ab Frontier, Seoul, Korea). ImageQuant LAS-4000 Mini luminescence image analyzer (LAS-4000, Fujifilm Life Sciences, Tokyo, Japan) was used to expose the chemiluminescent samples and capture digital images, which were analyzed using ImageJ software (National Institutes of Health, Bethesda, MD, USA).

### 4.10. Statistical Analyses

Data are presented as numbers (percentages) or means ± standard deviations. Data sets were probed for normality using Kolmogorov–Smirnov test. Repeated-measures analysis of variance and subsequent post hoc analysis by Student’s *t*-test or Mann–Whitney U test was performed with Bonferroni’s correction to account for multiple comparisons. For non-parametric independent variables, including the datasets from the ex vivo study, data are presented as median + interquartile range, and Mann–Whitney U test was used for data analysis. A *p*-value < 0.05 was considered significant. All statistical analyses were performed using SPSS version 25.0 (IBM Corp., Armonk, NY, USA).

## Figures and Tables

**Table 1:** Primer lists.

Target Gene	Primer Sequences (5′–3′)
*MITF*	Forward: GGCTTGATGGATCCTGCTTTGC Reverse: GAAGGTTGGCTGGACAGGAGTT
*Tyrosinase*	Forward: GCACAGATGAGTACATGGGAGG Reverse: CTGATGGCTGTTGTACTCCTCC
*TYRP-1*	Forward: TCTCAATGGCGAGTGGTCTGTG Reverse: CCTGTGGTTCAGGAAGACGTTG
*p16*	Forward: CTCGTGCTGATGCTACTGAGGA Reverse: GGTCGGCGCAGTTGGGCTCC
*p21*	Forward: AGGTGGACCTGGAGACTCTCAG Reverse: TCCTCTTGGAGAAGATCAGCCG
*p53*	Forward: CCTCAGCATCTTATCCGAGTGG Reverse: TGGATGGTGGTACAGTCAGAGC
*MMP-2*	Forward: AGCGAGTGGATGCCGCCTTTAA Reverse: CATTCCAGGCATCTGCGATGAG
*MMP-9*	Forward: GCCACTACTGTGCCTTTGAGTC Reverse: CCCTCAGAGAATCGCCAGTACT
*GAPDH*	Forward: TGAGGTCACGGACGATTACT Reverse: GTAGGCCCACGAAACAAATGAT

## Data Availability

Not applicable.
